# Chemical-induced disease relation extraction via attention-based distant supervision

**DOI:** 10.1186/s12859-019-2884-4

**Published:** 2019-07-22

**Authors:** Jinghang Gu, Fuqing Sun, Longhua Qian, Guodong Zhou

**Affiliations:** 1 Natural Language Processing Lab, School of Computer Science and Technology, Soochow University, 1 Shizi Street, Suzhou, China; 2 Big Data Group, Baidu Inc., Beijing, China; 3 Department of Gynecology Minimally Invasive Center, Beijing Obstetrics and Gynecology Hospital, Capital Medical University, Beijing, China

**Keywords:** Biomedical relation extraction, Distant supervision, Attention, Deep learning

## Abstract

**Background:**

Automatically understanding chemical-disease relations (CDRs) is crucial in various areas of biomedical research and health care. Supervised machine learning provides a feasible solution to automatically extract relations between biomedical entities from scientific literature, its success, however, heavily depends on large-scale biomedical corpora manually annotated with intensive labor and tremendous investment.

**Results:**

We present an attention-based distant supervision paradigm for the BioCreative-V CDR extraction task. Training examples at both intra- and inter-sentence levels are generated automatically from the Comparative Toxicogenomics Database (CTD) without any human intervention. An attention-based neural network and a stacked auto-encoder network are applied respectively to induce learning models and extract relations at both levels. After merging the results of both levels, the document-level CDRs can be finally extracted. It achieves the precision/recall/F1-score of 60.3%/73.8%/66.4%, outperforming the state-of-the-art supervised learning systems without using any annotated corpus.

**Conclusion:**

Our experiments demonstrate that distant supervision is promising for extracting chemical disease relations from biomedical literature, and capturing both local and global attention features simultaneously is effective in attention-based distantly supervised learning.

## Background

Chemical/Drug discovery is a complex and onerous process which is often accompanied by undesired side effects or toxicity [1]. To reduce the risk and speed up chemical development, automatically understanding interactions between chemicals and diseases has received considerable interest in various areas of biomedical research [2–4]. Such efforts are important not only for improving chemical safety but also for informing potential relationships between chemicals and pathologies [5]. Although many attempts [6, 7] have been made to manually curate amounts of chemical-disease relations (CDRs), this curation is still inefficient and can hardly keep up to date.

**Fig. 1:**
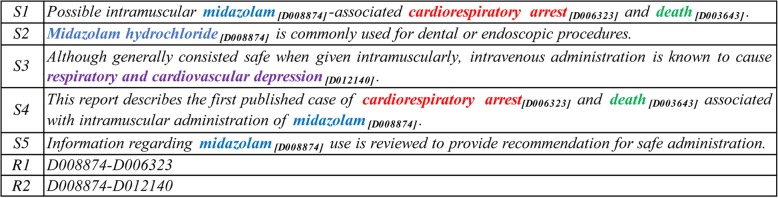
The title and abstract of the sample document (PMID: 2375138)

For this purpose, the BioCreative-V community for the first time proposed the challenging task of automatically extracting CDRs from biomedical literature [8, 9], which was intended to identify chemical-induced disease (CID) relations from PubMed articles. Different from previous well-known biomedical relation extraction tasks, such as protein-protein interaction [10, 11] and disease-gene association [12, 13], the BioCreative-V task required the output of the extracted document-level relations with entities normalized by Medical Subject Headings (MeSH) [14] identifiers. In other words, participants were asked to extract such a list in terms of <Chemical ID, Disease ID> pairs from the entire document. For instance, Fig. 1 shows the title and abstract of the document (PMID: 2375138) with two target CID relations, i.e. <D008874, D006323 > and < D008874, D012140>. The colored texts are chemicals and diseases with the corresponding subscripts of their MeSH identifiers, and same entities are represented in the same color.

Since relation extraction task can be cast as a classification problem, many supervised machine learning methods [15–23] have been investigated to extract CID relations. However, since supervised learning methods usually require a set of instance-level training data to achieve high performance, CID relations annotated at document level in the CDR corpus are not directly applicable and have to be transformed to relation instances for training classifiers. Erroneous relation instances are inevitable during this transformation [18], leading to flat F1-score around 60% without knowledge base features, in large part due to the small scale of the CDR corpus with only 1000 abstracts in the training and development sets totally.

Distant supervision (DS) provides a promising solution to the scarcity of the training corpora. It automatically creates training instances by heuristically aligning facts in existing knowledge bases to free texts. Mintz et al. [24] assumes that if two entities have a relationship in a known knowledge base, then all sentences that contain this pair of entities will express the relationship. Since its emergence, distant supervision has been widely adopted to information extraction in news domain [24] as well as in biomedical text mining [25–28]. However, the original assumption by Mintz et al. [24] does not always hold and false-positive instances may be generated during automatic instance construction procedure. The critical issue in distant supervision is, therefore, how to filter out these incorrect instances. Many methods have been proposed to tackle this problem [30–33] and show promising results in their respective settings, but few [26–28] have demonstrated superiority in performance over supervised ones on the benchmark corpora in the biomedical domain.

We present a distant supervision paradigm for the document-level CDR task and propose a series of ranking-based constraints in order to filtering out the noise of training instances generated by distant supervision. Specifically, intra- and inter-sentence training instances are first projected respectively from the CTD database. Then, a novel neural network integrated with an attention mechanism is applied to address the intra-sentence level relation extraction. The attention mechanism automatically allocates different weights to different instances, thus is able to selectively focus on relevant instances other than irrelevant ones. Meanwhile, a stacked auto-encoder neural network is used to extract the relations at inter-sentence level. Its encoder and decoder facilitate higher level representations of relations across sentences. Finally, the results at both levels are merged to obtain the CID relations between entities at document level. The experimental results indicate that our approach exhibits superior performance compared with supervised learning methods. We believe our approach is robust and can be used conveniently for other relation extraction tasks with less efforts needed for domain adaptation.

## Related works

Thanks to the availability of the BioCreative-V CDR corpus, researchers have employed various supervised machine learning methods to extract the CID relations, including conventional machine learning and deep learning.

Early studies only tackled the CID relation extraction at intra-sentence level using statistical models, such as the logistic regression model by Jiang et al. [15] and the Support Vector Machine (SVM) by Zhou et al. [16]. Lexical and syntactic features were used in their models. Later, the CID relation extraction at inter-sentence level is also considered. An integrated model combining two maximum entropy classifiers at intra- and inter-sentence levels respectively, is proposed by Gu et al. [17], where various linguistic features are leveraged. In addition to linguistic features, external knowledge resources are also exploited to improve performance. During the BioCreative-V official online evaluation, Xu et al. [19] achieved the best performance with two SVM classifiers at sentence and document levels, respectively. Rich knowledge-based features were fed into these two classifiers. Similar to Xu et al. [19], Pons et al. [20] and Peng et al. [21] also applied SVM models with knowledge features including statistical, linguistic, and various domain knowledge features for the CID relations. Additionally, a large amount of external training data was exploited in Peng et al. [21] as well.

Recently deep learning methods have been investigated to extract CID relations. Zhou et al. [22] used a Long Short-Term Memory (LSTM) network model together with an SVM model to extract the CID relations. The LSTM model was designed to abstract semantic representation in long range while the SVM model was meant to grasp the syntactic features. Gu et al. [23] proposed a Convolutional Neural Network (CNN) model to learn a more robust relation representation based on both word sequences and dependency paths for the CID relation extraction task, which could naturally characterize the relations between chemical and disease entities. However, both the traditional learning and deep learning methods suffer from the same problems of the scarcity of the CDR corpus and the noise brought about by the transformation from document-level relations to instance-level relations.

As an alternative to supervised learning, distant supervision has been examined and show promising results in biomedical text mining, mostly in Protein-Protein Interaction (PPI) extraction. Thomas et al. [27] proposed the use of trigger words in distant supervision, i.e., an entity pair of a certain sentence is marked as positive (related) if the database has information about their interaction and the sentence contains at least one trigger word. Experiments on 5 PPI corpora show that distant supervision achieves comparable performance on 4 of 5 corpora. Bobić et al. [26] introduced the constraint of “auto interaction filtering” (AIF): if entities from an entity pair both refer to the same real-world object, the pair is labeled as not interacting. Experiments on 5 PPI corpora show mixed results. Bobić and Klinger [25] proposed the use of query-by-committee to select instances instead. This approach was similar to the active learning paradigm, with a difference that unlabeled instances are weakly annotated, rather than by human experts. Experiments on publicly available data sets for detection of protein-protein interactions show a statistically significant improvement in F1 measure. Poon et al. [28] applied the multi-instance learning method [30] to extracting pathway interactions from PubMed abstracts. Experiments show that distant supervision can attain an accuracy approaching supervised learning results.

## Distant supervision

Multi-instance learning is an effective way to reduce noise in distant supervision [29–33] with the *at-least-one* assumption stating that in all of sentences that containing the same entity pair, there should be at least one sentence which can effectively support the relationship. Formally, for the triplet *r*(*e*_*1*_, *e*_*2*_), all the sentences that mention both *e*_*1*_ and *e*_*2*_ constitute a relation bag with the relation *r* as its label, and each sentence in the bag is called an instance. Suppose that there are *N* bags {*B*_*1*_, *B*_*2*_,⋯, *B*_*N*_} existing in the training set and the *i*-th bag contains *m* instances *B*_*i*_ = { ${b}_1^i$, ${b}_2^i$,⋯, ${b}_m^i$ } (*i* = 1,⋯, *N*). The objective of multi-instance learning is to predict the labels of unseen bags. It needs to first learn a relation extractor based on the training set and then predict relations for the test set by the learned relation extractor. Specifically, for a bag *B*_*i*_ in the training set, we need to extract features from the bag (from one or several valid instances) and then use them to train a classifier. For a candidate bag in the test set, we need to extract features in the same way and use the classifier to predict the relation between a given entity pair.

In order to alleviate the noise problem caused by distant supervision, we adopt an attention-based neural network model to automatically assign different weights to different instances. This approach is able to selectively focus on the relevant instances through assigning higher weights to relevant instances and lower weights to the irrelevant ones.

## Materials and methods

**Fig. 2:**
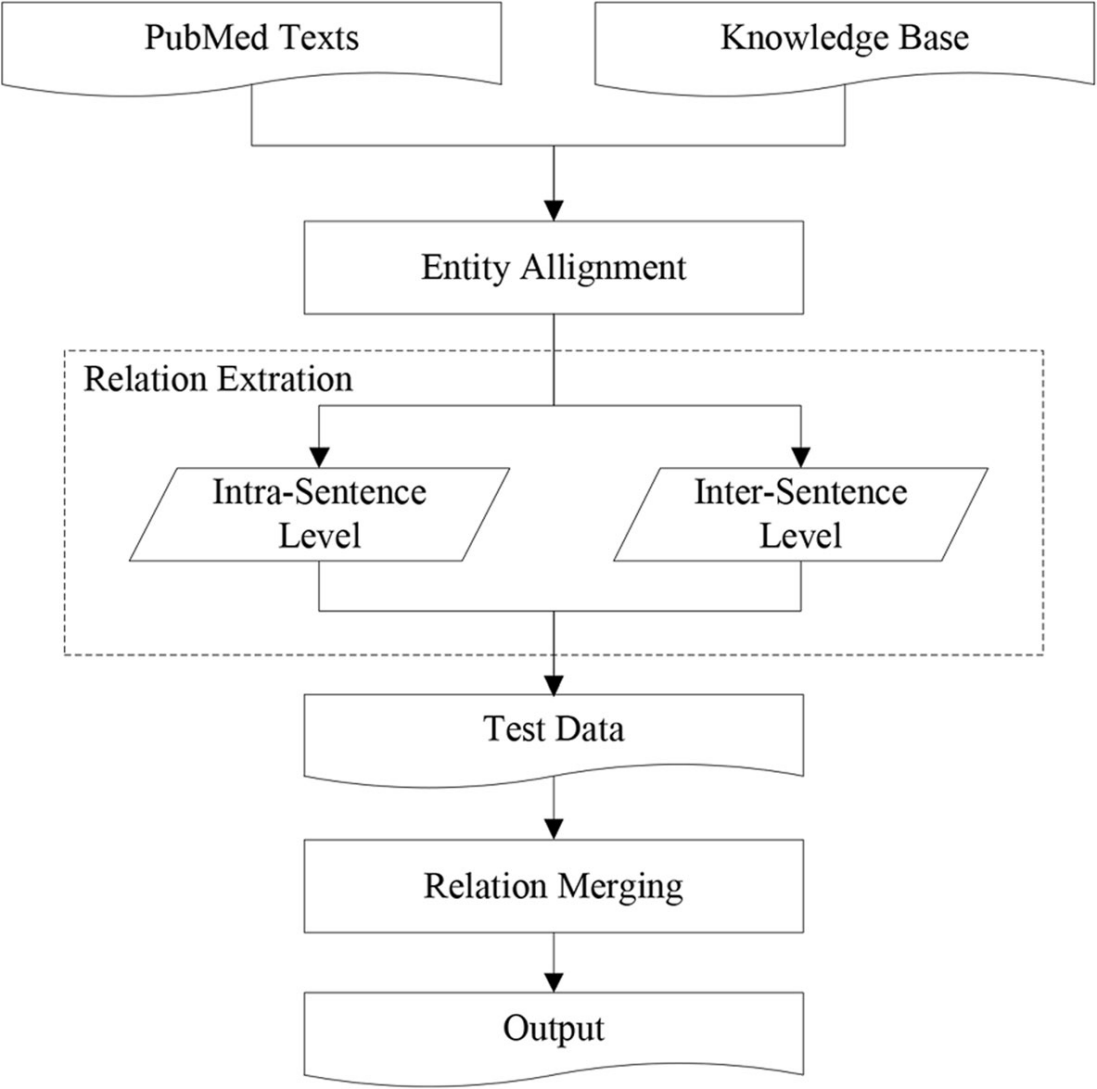
The system workflow diagram

Figure 2 illustrates the main architecture of our approach. We first heuristically align facts from a given knowledge base to texts and then use this alignment results as the training data to learning a relation extractor. We then conduct the relation extraction at two levels. For the intra-sentence level, we propose an instance-level attention-based model within a multi-instance learning paradigm. For the inter-sentence level, we propose a stacked auto-encoder neural network with simple and effective lexical features, which further improves the ensemble performance of the document-level CID relation extraction task. We finally merged the classification results from both levels to acquire the final document-level CID relations between entities.

The BioCreative-V CDR corpus composes of 1500 biomedical articles collected from MEDLINE database [8, 21] which are further split into three different datasets for training, developing and testing, respectively. All chemicals, diseases and CID relations in the corpus are manually annotated and indexed by MeSH concept identifiers, i.e., the relations were annotated in a document between entities rather than between entity mentions. It is important to note that since the official annotation results didn’t announce the inter-annotator agreement (IAA) of the CID relations, Wiegers et al. [34] reported an approximate estimate score of 77%. Table 1 reports the statistics on the numbers of articles and relations in the corpus.

**Table 1:** The CID relation statistics on the corpus

Task Datasets	# of Articles	# of CID Relations
Training	500	1038
Development	500	1012
Test	500	1066

In our distant supervision paradigm, the CTD database [6, 7] was used as the knowledge resource and its relation facts were aligned to the PubMed literature to construct training data. For fair comparison with other systems and maximal scale of training data, the entity alignment procedure was devised as follows:

i. Construct the PubMed abstract set (PubMedSet) according to the CTD database, from which the abstracts already annotated in the CDR corpus are removed;
ii. A named entity recognition and normalization process is conducted to identify and normalize the chemicals and diseases in the PubMedSet abstracts;
iii. For every abstract, if a chemical/disease pair is curated in the CTD database as the relation fact ‘Marker/Mechanism’, then the pair is marked as a positive CID relation, otherwise as a negative one.

For instance, the chemical-disease relational facts <D013752, D011559 > and < D013752, D009325 > curated in CTD can be aligned with the following discourse from the literature (PMID:10071902) which is collected into PubMedSet:

- ***Tetracyclines***_*[D013752]*_ have long been recognized as a cause of ***pseudotumor cerebri***_*[D011559]*_ in adults, but the role of ***tetracyclines***_*[D013752]*_ in the pediatric age group has not been well characterized in the literature and there have been few reported cases*.*
- We retrospectively analyzed the records of all patients admitted with a diagnosis of ***pseudotumor cerebri***_*[D011559]*_ who had documented usage of a ***tetracycline***_*[D013752]*_-class drug immediately before presentation at the Hospital For Sick Children in Toronto, Canada, from January 1, 1986, to March 1, 1996.
- Symptoms included headache (6 of 6), ***nausea***_*[D009325]*_ (5 of 6), and diplopia (4 of 6).

Among these texts, the relational fact <D013752, D011559 > totally co-occur three times in sentence a) and b), and the fact thus can generate an intra-sentence level relation bag with three instances inside, however, the 2nd occurrence doesn’t convey the relationship, therefore it is a false positive. Differently, the relational fact < D013752, D009325 > has no co-occurrence within a single sentence, the nearest mentions of chemical ***tetracycline*** and disease ***nausea*** thus generate the relation instance to form an inter-sentence level relation bag. In a similar way, this paradigm of distant supervision can be extended to other relation extraction tasks as well, such as PPI/DDI (Protein-Protein Interaction/Drug-Drug interaction) extraction [26, 27] and pathway extraction [28].

Note that excluding the CDR abstracts from PubMedSet is important because involvement of any CDR abstracts would either reuse the CDR training set or overfit our models for the CDR test set, thus diminishing the strength of distant supervision.

Table 2 reports the statistics on the final generated training set, which contains ~ 30 K PubMed abstracts with ~ 9 K chemicals and over 3 K diseases, between which more than 50 K positive relations are obtained, including both intra- and inter-sentence levels. The sheer size of the training set is remarkable since manually labeling such big corpus would be a daunting task.

**Table 2:** Statistics on the generated training set

Types	Count
PMIDs	30,884
Chemical Entities	9113
Chemical Mentions	358,395
Disease Entities	3525
Disease Mentions	267,196
Relations	54,729

## Intra-sentence relation extraction

**Fig. 3:**
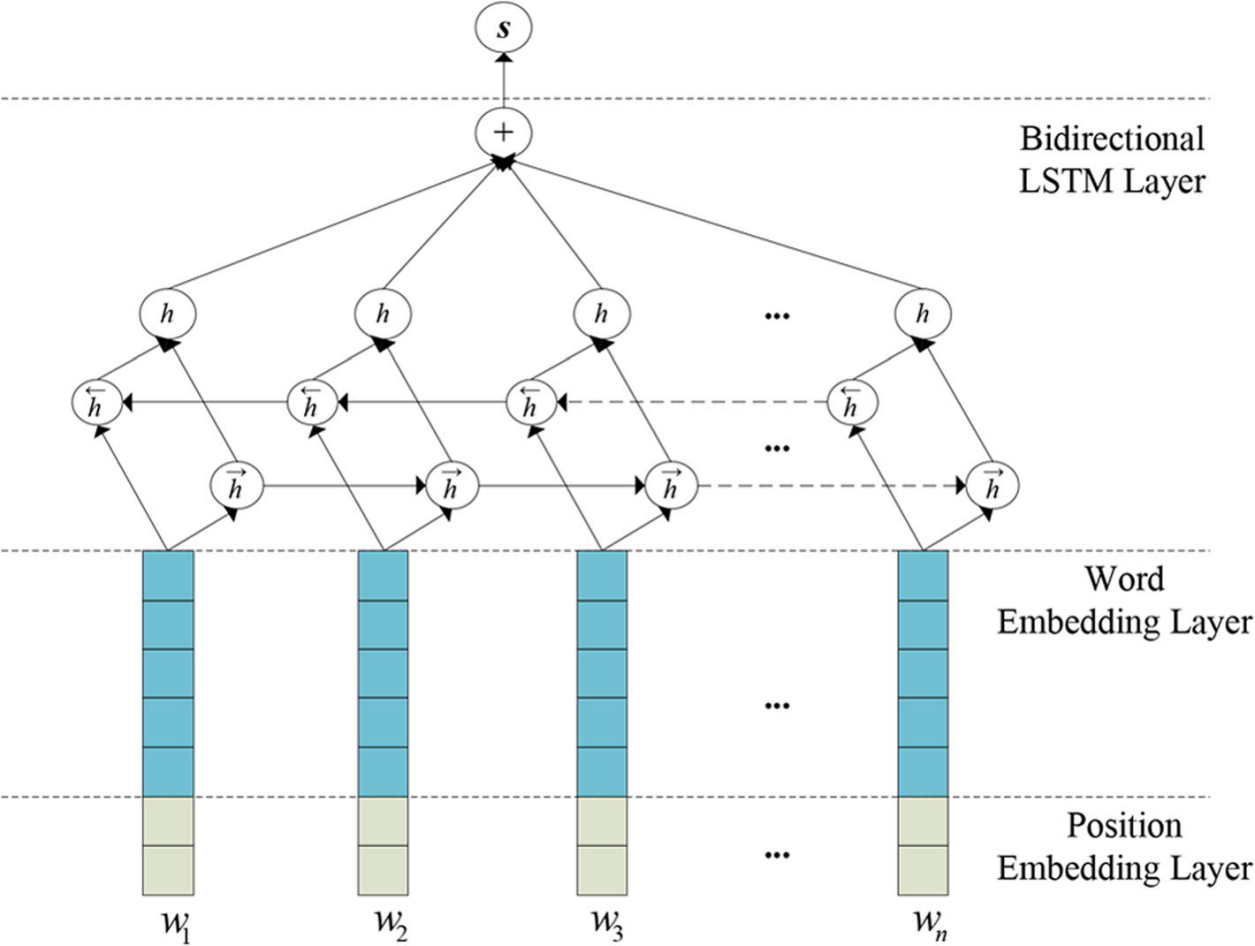
The architecture of the Instance Representation module

**Fig. 4:**
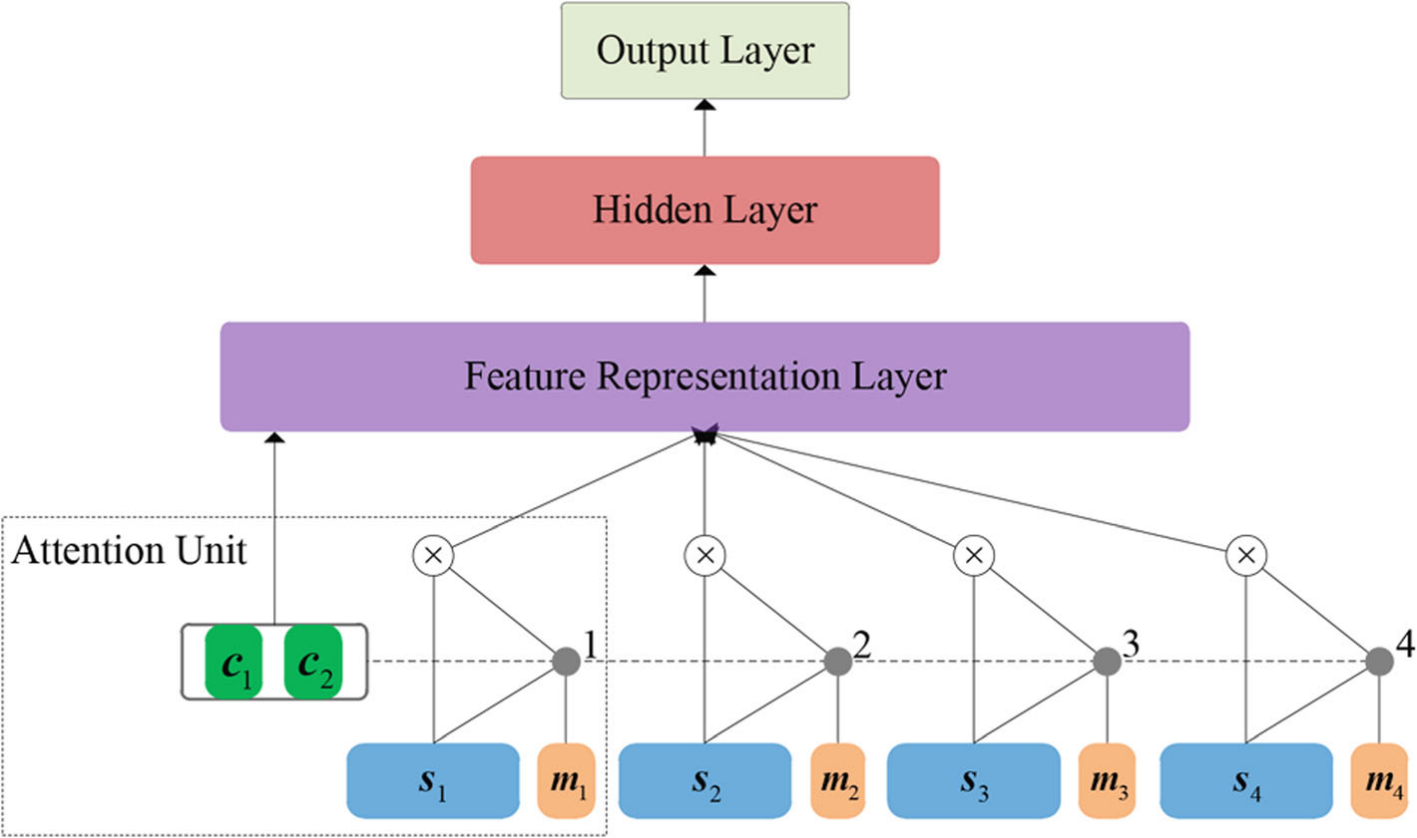
The architecture of the instance-level attention module

In our attention-based distant supervision approach for intra-sentence relation extraction, a relation is considered as a bag *B* of multiple instances in different sentences that contain the same entity pair. Thus, our attention-based model contains two hierarchical modules: the lower *Instance Representation Module* (Fig. 3) and the higher *Instance-Level Attention Module* (Fig. 4). The former aims to obtain the semantic representation of each instance within the bag, while the latter can measure the importance of each instance in the bag in order to integrate into the bag representation and thereby predicts the bag’s label.

### Instance Representation Module

Figure 3 illustrates the architecture of our Instance Representation Module consisting of two layers: *Embedding Layer* and *Bidirectional LSTM Layer*. The module takes as an input instance a sentence that contains a target entity pair and output a high-level representation vector. The words and their positions in the sentence are first mapped to low-dimensional real valued vectors called word embeddings [35] and position embeddings [36, 37] respectively. Then the two embeddings are concatenated into a joint embedding to represent each word. Finally, a recurrent neural network based on bidirectional LSTM is used to encode the sequence of joint embeddings.

#### Embedding Layer

The *Embedding Layer* is used to transform each word in the sentence into a fixed-length joint embedding concatenated by a word embedding and its position embedding. Word embeddings are encoded in terms of column vectors in an embedding matrix $\boldsymbol{T}\in {\mathbf{\mathbb{R}}}^{d_T\times \mid {V}_T\mid }$, where *d*_*T*_ is the dimension of the word embeddings and |*V*_*T*_| is the size of the vocabulary. Thus, the word embedding ***w***_*i*_ for a word *w*_*i*_ can be obtained using matrix-vector product as follows:

1 $$ {\boldsymbol{w}}_i={\boldsymbol{Tu}}^{w_i} $$

where the vector ${\boldsymbol{u}}^{w_i}$ has the value of 1 at index *w*_*i*_ and zeroes otherwise. The parameter ***T*** **is** the vocabulary table to be learned during training, while the hyper-parameter *d*_*T*_ is the word embedding dimension.

Position embeddings [36] encode the information about the relative distance of each word to the target chemical and disease respectively, and they are also encoded by column vectors in an embedding matrix $\boldsymbol{P}\in {\mathbf{\mathbb{R}}}^{d_P\times \mid {V}_P\mid }$, where |*V*_*P*_| is the size of vocabulary and *d*_*P*_ is a hyper-parameter referring to the dimension of the position embedding. We use ${\boldsymbol{p}}_i^c$ and ${\boldsymbol{p}}_i^d$ to represent the position embeddings of each word to the target chemical and disease respectively.

After obtaining the word embedding ***w***_*i*_ and the position embeddings ${\boldsymbol{p}}_i^c$ and ${\boldsymbol{p}}_i^d$, we concatenate these vectors into a single vector ***t***_*i*_ as the joint embedding of the word.

2 $$ {\boldsymbol{t}}_i=\left[{\boldsymbol{w}}_i;{\boldsymbol{p}}_i^c;{\boldsymbol{p}}_i^d\right] $$

#### Bidirectional LSTM Layer

Recurrent Neural Networks (RNNs) are promising deep learning models that can represent a sequence of arbitrary length in a vector space of a fixed dimension [38–40]. We adopt a variant of bidirectional LSTM models introduced by [41], which adds weighted peephole connections from the Constant Error Carousel (CEC) to the gates of the same memory block.

Typically, an LSTM-based recurrent neural network consists of the following components: an input gate ***i***_*t*_ with corresponding weight matrix ***W***^(*i*)^, ***U***^(*i*)^ and ***b***^*(i)*^; a forget gate ***f***_*t*_ with corresponding weight matrix ***W***^(*f*)^, ***U***^(*f*)^ and ***b***^*(f)*^; an output gate ***o***_*t*_ with corresponding weight matrix ***W***^(*o*)^, ***U***^(*o*)^ and ***b***^*(o)*^. All these gates use the current input ***x***_*t*_ and the state ***h***_*i*-1_ that the previous step generated to decide how to take the inputs, forget the memory stored previously, and output the state generated later. These calculations are illustrated as follows:

3 $$ {\boldsymbol{i}}_t=\sigma \left({\boldsymbol{W}}^{(i)}\cdot {\boldsymbol{x}}_t+{\boldsymbol{U}}^{(i)}\cdot {\boldsymbol{h}}_{t-1}+{\boldsymbol{b}}^{(i)}\right) $$

4 $$ {\boldsymbol{f}}_t=\sigma \left({\boldsymbol{W}}^{(f)}\cdot {\boldsymbol{x}}_t+{\boldsymbol{U}}^{(f)}\cdot {\boldsymbol{h}}_{t-1}+{\boldsymbol{b}}^{(f)}\right) $$

5 $$ {\boldsymbol{o}}_t=\sigma \left({\boldsymbol{W}}^{(o)}\cdot {\boldsymbol{x}}_t+{\boldsymbol{U}}^{(o)}\cdot {\boldsymbol{h}}_{t-1}+{\boldsymbol{b}}^{(o)}\right) $$

6 $$ {\boldsymbol{u}}_t=\tanh \left({\boldsymbol{W}}^{(g)}\cdot {\boldsymbol{x}}_t+{\boldsymbol{U}}^{(g)}\cdot {\boldsymbol{h}}_{t-1}+{\boldsymbol{b}}^{(g)}\right) $$

7 $$ {\boldsymbol{c}}_t={\boldsymbol{i}}_t\otimes {\boldsymbol{u}}_t+{\boldsymbol{f}}_t\otimes {\boldsymbol{c}}_{t-1} $$

where *σ* denotes the logistic function, ⊗ denotes element-wise multiplication, ***W***^(*)^ and ***U***^(*)^ are weight matrices, and ***b***^(*)^ are bias vectors. The current cell state ***c***_*t*_ will be generated by calculating the weighted sum using both previous cell state and the information generated by the current cell [41]. The output of the LSTM unit is the hidden state of recurrent networks, which is computed by Eq. (7) and is passed to the subsequent units:

8 $$ {\boldsymbol{h}}_t={\boldsymbol{o}}_t\otimes \tanh \left({\boldsymbol{c}}_t\right) $$

We use a bidirectional LSTM network to obtain the representation of sentences since the network is able to exploit more effective information both from the past and the future. For the *i*-th word in the sentence, we concatenate both forward and backward states as its representation as follows:

9 $$ {\boldsymbol{h}}_i=\left[{\boldsymbol{h}}_i^f;{\boldsymbol{h}}_i^b\right] $$

where ${\boldsymbol{h}}_i^f$ is the forward pass state and ${\boldsymbol{h}}_i^b$ is the backward pass state. Finally an average operation is performed to run over all the LSTM units to obtain the representation of the relation instance ***s***_*j*_:

10 $$ {\boldsymbol{s}}_j=\frac{1}{n}\sum \limits_{i=1}^n{\boldsymbol{h}}_i $$

### Instance-Level Attention Module

Figure 4 presents the architecture of our attention-based model which includes four parts: *Attention Unit*, *Feature Representation Layer*, *Hidden Layer* and *Output Layer*. The attention model is supposed to effectively adjust the importance of the different instances within a relation bag, i.e., the more reliable the instance is, the larger weight it will be given. In this way the model can selectively focus on those relevant instances.

#### Attention Unit

The attention unit is designed for calculating the weights of different instances. In order to incorporate more semantic information of instances, our attention unit introduces *Location Embedding*, *Concept Embedding* and *Entity Difference Embedding* for weight calculation.

##### Location Embedding

Since instances are usually located at different positions in the literature, such as title and abstract, we believe that the location information is of great significance for determining the importance of instances in a relation bag. Therefore, *Location Embedding* is designed to capture the relative location feature of each instance. Location embeddings are encoded in terms of column vectors in an embedding matrix $\boldsymbol{L}\in {\mathbf{\mathbb{R}}}^{d_L\times \mid {V}_L\mid }$, where *d*_*L*_ is the dimension of the location embeddings and |*V*_*L*_| is the size of the vocabulary. Specifically, in our work, four different location markers are used to represent the location information of each instance as shown in Table 3:

**Table 3:** Feature names and their locations

Name	Location
T	At the title.
A_Fst	At the first sentence of the abstract.
A_Lst	At the last sentence of the abstract.
A_Mdl	In the middle of the abstract.

##### Concept Embedding

In order to incorporate more semantic information of entities, we use *Concept Embedding* to represent entities, which consists of entity identifier embeddings and hyponymy embeddings.

Identifier embeddings encode entity identifiers into low-dimensional dense vectors and are encoded in terms of column vectors in an embedding matrix $\boldsymbol{E}\in {\mathbf{\mathbb{R}}}^{d_E\times \mid {V}_E\mid }$, where *d*_*E*_ is the dimension of the identifier embeddings and |*V*_*E*_| is the size of the vocabulary.

Previous research [18, 23] has found that the hypernym/hyponym relationship between entities also improve the performance of relation extraction. We use a binary hyponym tag to determine whether an entity is most specific in the document according to the MeSH tree numbers of each entity identifier. We then convert the hyponym tag into low-dimensional dense vector as its hyponym embeddings. Hyponym embeddings are encoded by column vectors as well in an embedding matrix $\boldsymbol{Q}\in {\mathbf{\mathbb{R}}}^{d_Q\times \mid {V}_Q\mid }$, where *d*_*Q*_ is the dimension of the hyponym embeddings and |*V*_*Q*_| is the size of the vocabulary. After obtaining the identifier embedding ***e***_*i*_ and the hyponym embedding ***q***_*i*_, the concept embedding ***c***_*i*_ is generated by concatenating these two vectors as follows:

11 $$ {\boldsymbol{c}}_i=\left[{\boldsymbol{e}}_i;{\boldsymbol{q}}_i\right] $$

##### Entity Difference Embedding

Recently, many knowledge learning approaches regard the relation between entities as a translation problem and achieve the state-of-the-art prediction performance [42–44]. The basic idea behind these models is that, the relationship *r* between two entities corresponds to a translation from the head entity *e*_1_ to the tail entity *e*_2_, that is, ***e***_1_ + **r** ≈ ***e***_2_ (the bold, italic letters represent the corresponding vectors). Motivated by these findings, we also use the difference value between the concept embeddings of *e*_1_ and *e*_2_ to represent the target relation between them:

12 $$ \boldsymbol{r}={\boldsymbol{c}}_1-{\boldsymbol{c}}_2 $$

##### Bag Representation

According to [45], the semantic representation of bag *S* for a certain pair of entities relies on the representations of all its instances, each of which contains information about whether, and more precisely the probability that, the entity pair holds the relation in that instance. Thus, we calculated the weighted sum of instances contained in bag *S* to obtain the bag representation.

Suppose a given relation bag *S* contains *m* instances, i.e., *S* = {*s*_1_, *s*_2_, …, *s*_*m*_}, then the representation of *S* can be defined as:

13 $$ \boldsymbol{u}=\sum \limits_{k=1}^n{\alpha}_k{\boldsymbol{s}}_k $$

where ***s***_*k*_ is the instance representation and *α*_*k*_ is its attention weight. We argue that the weight is highly related to the instance representation, the instance location and the entity difference embedding, thus, we calculate *α*_*k*_ as follows:

14 $$ {\alpha}_k=\frac{\exp \left(\Gamma \left({\boldsymbol{s}}_k,{\boldsymbol{m}}_k,\boldsymbol{r}\right)\right)}{\sum \limits_l\exp \left(\Gamma \left({\boldsymbol{s}}_l,{\boldsymbol{m}}_l,\boldsymbol{r}\right)\right)} $$

where Г(∙) is a measure function that reflects the relevance between each instance and corresponding relation *r* and is defined as:

15 $$ \Gamma \left({\boldsymbol{s}}_k,{\boldsymbol{m}}_k,\boldsymbol{r}\right)={\boldsymbol{v}}^{\boldsymbol{T}}\tanh \left({\boldsymbol{W}}_s\cdot {\boldsymbol{s}}_k+{\boldsymbol{W}}_m\cdot {\boldsymbol{m}}_k+{\boldsymbol{W}}_r\cdot \boldsymbol{r}+{\boldsymbol{b}}_s\right) $$

where ***s***_*k*_, ***m***_*k*_ are the instance representation and location embedding respectively, and ***r*** is the entity difference embedding defined in Eq. (12) while ***W***_***s***_, ***W***_***m***_ and ***W***_***r***_ are respective weight matrices, ***b***_***s***_ is the bias vector, and ***v***^T^ is the weight vector. Through Eqs. (13) to (15), an instance-level attention mechanism can measure and allocate different weights to different instances, thus give more weights to true positive instances and less weights to wrongly labeled instances to alleviate the impact of noisy data.

#### Feature Representation Layer

The bag representation and the chemical/disease embeddings are conjoined to produce the feature vector ***k*** **= [*c***_***1***_;***c***_***2***_;***u*]** as the input to the hidden layer.

#### Hidden Layer

In the hidden layer, both Linear and non-linear operations are applied in order to convert the vector ***k*** to the final representation ***z*** as follows:

16 $$ \boldsymbol{z}=\tanh \left({\boldsymbol{W}}_1\boldsymbol{k}+{\boldsymbol{b}}_1\right) $$

Note that, a dropout operation is performed on vector ***z*** during the training process to mitigate the over-fitting issue. However, no dropout operation on ***z*** is needed during the testing process.

#### Softmax Layer

The softmax layer which takes as input the vector ***z*** calculates each instance confidence of the relations:

17 $$ \boldsymbol{o}=\mathrm{soft}\max \left({\boldsymbol{W}}_2\boldsymbol{z}+{\boldsymbol{b}}_2\right) $$

where the vector ***o*** denotes the final output, each dimension of which represents the probability that the instance belongs to a specific relationship.

The following objective function is then adopted in order to learn the network parameters, which involves the vector ***o*** together with gold relation labels in the training set:

18 $$ \boldsymbol{J}\left(\theta \right)=-\frac{1}{m}\sum \limits_{i=1}^m\log p\left({y}_i|{x}_i,\theta \right)+\lambda {\left\Vert \theta \right\Vert}^2 $$

where the gold label *y*_*i*_ corresponds to the training relation bag *x*_*i*_ and *p*(*y*_*i*_|*x*_*i*_,*θ*) thus denotes the probability of *y*_*i*_ in the vector ***o***, *λ* denotes the regularization factor and *θ* = {***T***, ***E***, ***Q***, ***W***_*s*_, ***W***_*m*_, ***W***_*r*_, ***b***_*s*_, ***v***, ***W***_1_, ***b***_1_, ***W***_2_, ***b***_2_} is the parameter set.

## Inter-sentence relation extraction

**Fig. 5:**
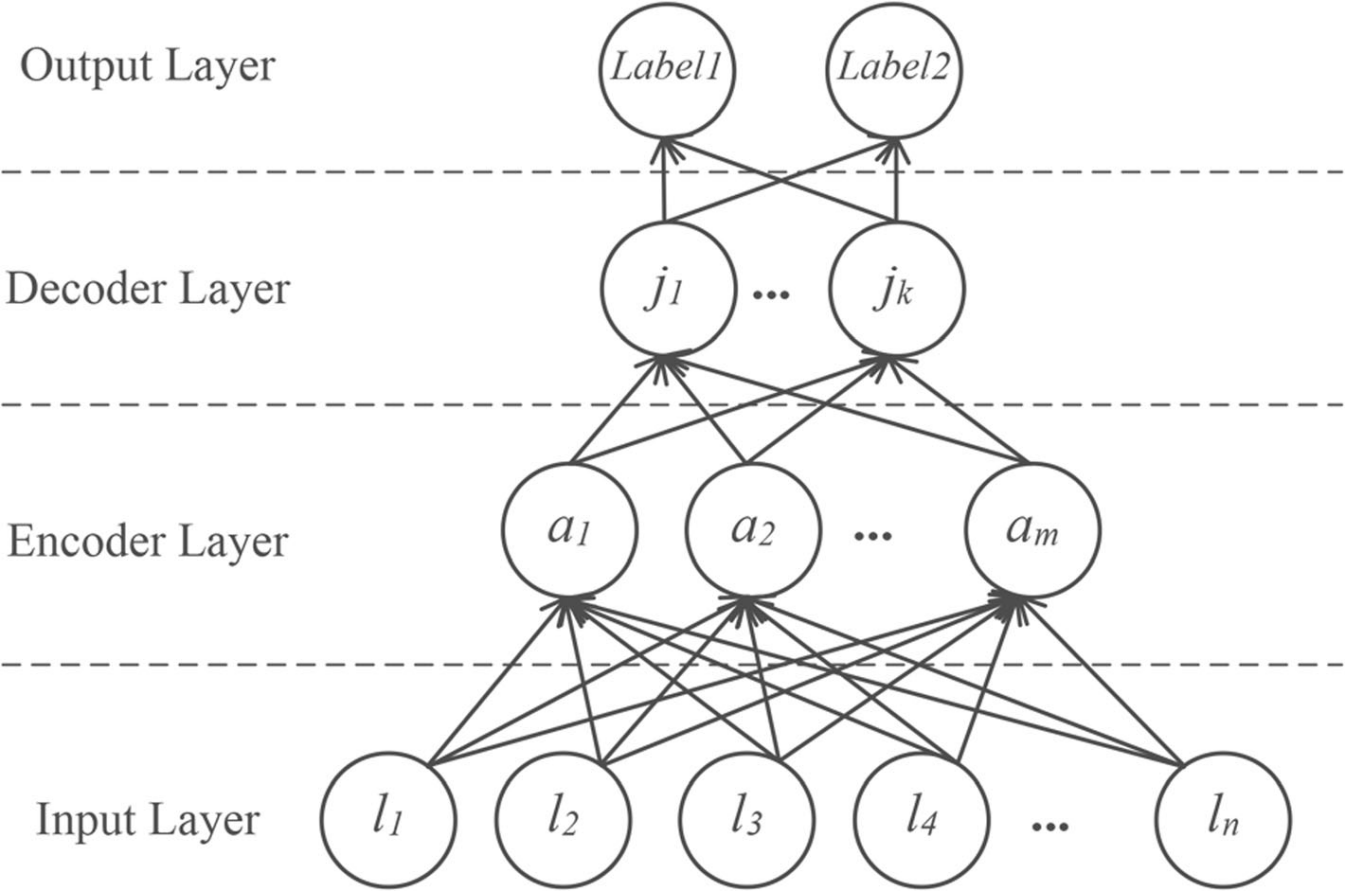
The stacked auto-encoder neural network

Different from intra-sentence relations, an inter-sentence relation spans multiple sentences, it is, therefore, difficult to find a unified text span containing an entity pair. We thus propose a simple and effective stacked auto-encoder neural network with entity lexical features. Figure 5 depicts the structure of our stacked auto-encoder model which consists of four components: *Input Layer*, *Encoder Layer*, *Decoder Layer* and *Output Layer*.

### Input Layer

We take as the input the lexical features of an entity pair, including the word embeddings of entity mentions, the concept embeddings and the frequency embeddings of two entities. These embeddings are concatenated into the feature vector ***l***, which is then fed into the encoder layer.

For entity mentions, an embedding matrix $\boldsymbol{D}\in {\mathbf{\mathbb{R}}}^{d_D\times \mid {V}_D\mid }$ is used to convert the entity mentions into word embeddings through a look-up operation, where *d*_*D*_ is the dimension of the word embeddings and |*V*_*D*_| is the size of the vocabulary. If an entity has multiple mentions, then we use average operation to obtain the final representation vector of mentions.

Similar to intra-sentence relation extraction, the embedding matrices $\boldsymbol{F}\in {\mathbf{\mathbb{R}}}^{d_F\times \mid {V}_F\mid }$ and $\boldsymbol{G}\in {\mathbf{\mathbb{R}}}^{d_G\times \mid {V}_G\mid }$ are used to acquire two parts of the concept embeddings, i.e., the identifier embedding and the hyponym embedding, where *d*_*F*_ and *d*_*G*_ are the dimension of embeddings while |*V*_*F*_| and |*V*_*G*_| are the size of two vocabularies, respectively.

Finally, we calculate the frequency of entities and use an embedding matrix $\boldsymbol{M}\in {\mathbf{\mathbb{R}}}^{d_M\times \mid {V}_M\mid }$ to convert the frequencies into embeddings as well.

### Encoder Layer

The encoder layer applies linear and non-linear transformations on the feature vector ***l*** to obtain the higher-level feature vector ***a*** and defined as follows:

19 $$ \mathbf{a}=\tanh \left({\mathbf{W}}_{\mathbf{3}}\mathbf{l}+{\mathbf{b}}_{\mathbf{3}}\right) $$

### Decoder Layer

The decoder layer applies linear and non-linear transformations as well to obtain the higher-level feature vector ***j*** and defined as follows:

20 $$ \mathbf{j}=\tanh \left({\mathbf{W}}_{\mathbf{4}}\mathbf{a}+{\mathbf{b}}_{\mathbf{4}}\right) $$

As in the hidden layer in intra-sentence relation extraction, a dropout operation is performed on ***j*** during training while no dropout during testing.

### Softmax Layer

Similar to intra-sentence relation extraction, the vector ***j*** is routed into the softmax layer to produce the final output vector ***o,*** which contains the probability for each relation type.

21 $$ \boldsymbol{o}=\mathrm{softmax}\left({\boldsymbol{W}}_5\boldsymbol{j}+{\boldsymbol{b}}_5\right) $$

Likewise, the same objective function as in intra-sentence relation extraction is used to train the network:

22 $$ \boldsymbol{J}\left(\theta \right)=-\frac{1}{m}\sum \limits_{i=1}^m\log p\left({y}_i|{x}_i,\theta \right)+\lambda {\left\Vert \theta \right\Vert}^2 $$

where the gold label *y*_*i*_ corresponds to the training instance *x*_*i*_ and *θ* = {***D***, ***F***, ***G***, ***M***, ***W***_3_, ***b***_3_, ***W***_4_, ***b***_4_, ***W***_5_, ***b***_5_} is the set of parameters.

After the relation extraction at both intra- and inter-sentence levels, their results are merged to generate the final document-level CID relations between chemicals and diseases.

## Results

In this section, we first present our experiment settings, then we systematically evaluate the performance of our approach on the corpus.

### Experiments settings

We use the PubMedSet corpus constructed through the entity alignment as the training data to induce the models and randomly select one tenth of the training data as the development data to tune the parameters. After training, the extraction model is used to extract the CID relations on the test dataset of the CDR corpus. In addition, we preprocess the training corpus using the following steps:

- Remove characters that are not in English;
- Convert all uppercase characters into lowercase letters;
- Replace all numbers with a unified symbol;
- Use TaggerOne [46] to recognize and normalize the chemicals and diseases.

The RMSprop [47] algorithm was applied to fine-tune the model parameters. GloVe [48] was used to initialize the look-up Tables ***T*** and ***D***. Other parameters in the model were initialized randomly. Table 4 shows the details of the hyper-parameters for both attention-based model and stacked auto-encoder model.

**Table 4:** Hyper-parameters for two models

Method	Hyper-parameter	Value
Attention-based Model	Learning rate	0.004
	LSTM hidden state dimension	200
	Mini-batch size	500
	Word embedding dimension	300
	Position embedding dimension	50
	Identifier embedding dimension	100
	Hyponym embedding dimension	50
	Location embedding dimension	50
	Hidden layer nodes	250
	Dropout rate	0.3
Stacked Auto-encoder Model	Learning rate	0.008
	Mini-batch size	400
	Word embedding dimension	300
	Identifier embedding dimension	100
	Hyponym embedding dimension	50
	Encoder layer nodes	250
	Decoder layer nodes	50
	Dropout rate	0.3

All experiments were evaluated by the commonly used metrics Precision (P), Recall (R) and harmonic F-score (F).

### Experimental results

For comparison, we fine-tuned an intra-sentence level Hierarchical Recurrent Neural Network (Intra_HRNN) as the baseline system. Specifically, the baseline system used two fine-tuned bidirectional LSTM layers to extract relations. The first bidirectional LSTM layer, which is used to obtain the representations of instances, is the same with the attention model. The second bidirectional LSTM layer is used to obtain the representations of relation bags without attention. Table 5 shows the intra-sentence level performance of Intra_HRNN and our attention model (Intra_Attention) on the test set with gold standard entity annotations, respectively. The ablation tests were also performed with one of the four features removed when calculating attention weights.

**Table 5:** The performance of the Attention-based model on the test dataset at intra-sentence level

Methods	P(%)	R(%)	F(%)
Intra_HRNN (Baseline)	62.0	55.2	58.4
Intra-Attention	62.2	59.5	60.8
- Descriptor Embedding	61.1	54.2	57.5
- Hyponym Embedding	61.7	56.6	59.0
- Location Embedding	61.9	56.7	59.2
- Entity Difference Embedding	62.1	56.9	59.4

From the table, we can observe that:

- The F1 score of the baseline system Intra_HRNN can reach 58.4%, indicating that the HRNN structure can well integrate the overall information to capture the internal abstract characteristics of entity relations. However, when using the attention-based distant supervision, the F1 score at intra-sentence level can finally reach as high as 60.8%. This suggests that the attention mechanism can effectively evaluate the importance of different instances and represent the features of the relation bag.
- Among all the features, when the identifier embeddings is separated from the feature set, the system performance drops significantly and the F1 score is only 57.5%. This suggests that the identifier embeddings can reflect effective semantic information behind entities. Likewise, other three embeddings also contribute to improve the performance. The experimental results indicate that these features are complementary to each other when performing relation extraction at intra-sentence level.

Similar to intra-sentence level, we also used fine-tuned an inter-sentence level Hierarchical Recurrent Neural Network (Inter_HRNN) as the baseline system to replace the stacked auto-encoder model. Table 6 shows the performance of the baseline system and our Stacked Auto-encoder approach (Stacked_Autoencoder), respectively.

**Table 6:** The performance of the Stacked Auto-Encoder model on the test dataset at inter-sentence level

Methods	P(%)	R(%)	F(%)
Inter_HRNN (Baseline)	27.0	19.8	22.8
Stacked_Autoencoder	55.7	14.2	22.6

As shown in the table, the performance at inter-sentence level is relatively low. This indicates that the expressions of relations across sentences are complex and diverse, therefore it is hard to capture effective semantic information between two involved entities across sentences. When only taking inter-sentential relation into consideration, the F1 score of the baseline system Inter_HRNN can reach 22.8%, while the performance of our stacked auto-encoder network could reach 22.6%. However, compared with the baseline system, though the stacked auto-encoder model has a relatively lower recall, it has a significant advantage in precision.

After extracting relations at both levels, we merge the results to obtain the final document level CID relations. We investigated four combinations of the above different various intra-sentence and inter-sentence models and show in Table 7 the overall performance of the CID relation extraction on the test set using gold entity annotations.

**Table 7:** The overall performance on the test dataset

Methods	P(%)	R(%)	F(%)
① Intra_HRNN + Inter_HRNN	46.5	75.0	57.4
② Intra_HRNN + Stacked_Autoencoder	58.2	71.6	64.2
③ Intra_Attention + Inter_HRNN	46.9	79.3	59.0
④ Intra_Attention + Stacked_Autoencoder	60.3	73.8	66.4

It can be found from the table that the overall extraction performance of ‘Intra_HRNN + Inter_HRNN’ is relatively low, of which the F1 score can only reach 57.4%. Our approach ‘Intra_Attention + Stacked_Autoencoder’ obtained the best performance, with the F1 score as high as 66.4%. In addition:

- Methods with ‘Intra_Attention’ outperform ones with ‘Intra_HRNN’ by ~ 2 units of F1 as comparison of ③ with ① and ④ with ②. This is consistent with the performance improvement reported in Table 5, justifying the intra-level attention mechanism which effectively considers the importance of different instances in a relation bag.
- Methods with ‘Stacked_Autoencoder’ dramatically outperform ones with ‘Inter_HRNN’ by ~ 7 units of F1 as comparison of ② with ① and ④ with ③. Interestingly, for only inter-sentence evaluation in Table 6, though the two models maintain comparable F1-scores, ‘Stacked_Autoencoder’ drastically improves the performance of precision. This boost of precision enables ‘Stacked_Autoencoder’ to eliminate more false inter-sentence positive instances than ‘Inter_HRNN’, leading to higher overall precision, and thus more balanced F1-scores.

**Fig. 6:**
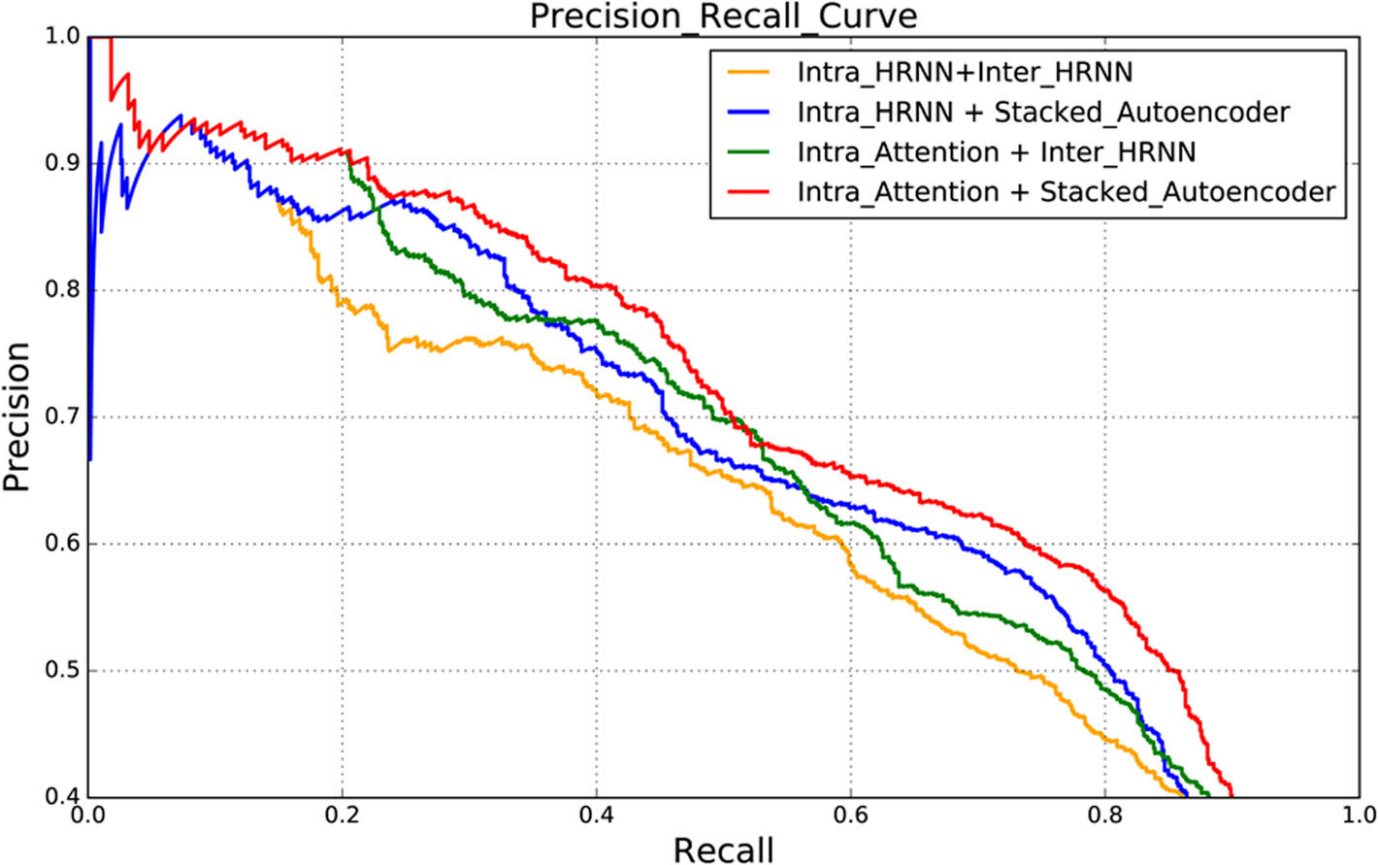
The precision-recall curve of different combinations

Figure 6 further compares the Precision-Recall curves of the four different combinations mentioned above. As is depicted in the figure, the curve of our model (i.e. “Intra_Attention + Stacked_Autoencoder”) is superior to other models, which shows a higher precision along with the recall. This suggests our distant supervision model can effectively extract the document level CID relations.

## Discussion

In this section, error analysis is first presented and then the comparison with other state-of-the-art systems is given.

### Error analysis

After careful examination of the experimental results, we classified the errors into four categories as follows:

- Complex expressions: if the instances in a certain relation bag fails to clearly express the corresponding CID relation, our distant supervision paradigm is unable to extract the relation correctly.
- Imprecise location information: in the intra-sentence level relation extraction, the location information of some unreliable instances would degrade the performance of our attention-based approach.
- Limited information on discourse: the inter-sentence relations are usually expressed through discourse and co-reference. In addition to conventional intra-sentence linguistic features, discourse analysis features derived from discourse parsing should be acquired to extract inter-sentence relations.
- Manual annotation disagreement: our investigation reveals that some extracted relations are considered as false positive, but actually should be true positive. These errors may arise from the fact that the IAA of the relation annotation is relatively low which is described in section *Materials*.

### Comparison with related works

We compare our work with the relevant works [17, 19–23, 49] in Table 8, which reports the performance of each system on the test dataset using gold standard entity annotations. We roughly divide these methods into four groups: rule-based, machine learning (ML) without additional resources, machine learning using external knowledge bases (KBs) and distant supervision.

**Table 8:** Comparisons with the related works

Methods	Systems	Description	P(%)	R(%)	F1(%)
Distant Supervision	Ours	Intra_Attention	62.2	59.5	60.8
		Intra_Attention + Stacked_Autoencoder	60.3	73.8	66.4
ML without KB	Gu et al. 2016 [17]	Intra_ME	60.4	50.3	54.9
		Intra_ME + Inter_ME	62.0	55.1	58.3
	Gu et al. 2017 [23]	CNN	59.7	55.0	57.2
		CNN + Inter_ME + PP	55.7	68.1	61.3
	Zhou et al. 2016 [22]	LSTM + SVM	64.9	49.3	56.0
		LSTM + SVM + PP	55.6	68.4	61.3
ML with KB	Ours	Intra_Attention + Stacked_Autoencoder + KBs	67.9	77.0	72.1
	Xu et al. 2016 [19]	SVM + KBs	65.8	68.6	67.2
	Pons et al. 2016 [20]	SVM + KBs	73.1	67.6	70.2
	Peng et al. 2016 [21]	Extra training data + SVM + KBs	71.1	72.6	71.8
Rule-based	Lowe et al. 2016 [49]	Heuristic rules	59.3	62.3	60.8

In the table, it shows that the rule-based system [49] obtained a competitive performance with the F-score of 60.8%. However, their construction process of the hand-crafted rules is laborious and time-consuming.

Compared with the rule-based approach, machine learning methods have shown a promising capability of extracting CID relations. Zhou et al. [22] proposed a hybrid method which combined an LSTM network with a tree kernel-based SVM for the sentence-level CID relations. After employing heuristic rules in the post-processing (PP) stage their F1-score reached 61.3%. Gu et al. [17] proposed different maximum entropy models, i.e. Intra_ME and Inter_ME, for intra- and inter-level relation extraction, respectively. They leveraged various linguistic features to extract the CID relations and the final performance of their method reached as high as 58.3%. Gu et al. [23] proposed a convolution neural network model based on contextual and dependency information and the final F1 score of their method reached 61.3%. Compared with the above methods, our distant supervision can automatically expand the size of training data through a weakly annotating procedure and obtain more relevant representations of relations, it therefore achieved the best performance with the F1 score of 66.4%. Particularly, our method promotes the intra-sentence performance significantly to the F1 score of 60.8%.

Among the systems using knowledge base [19–21], Peng et al. [21] extracted CID relations using an SVM model with rich features and augmented the training set with 18,410 external curated data in CTD, achieving the final F1 score as high as 71.8%. Similarly, Pons et al. [20] and Xu et al. [19] also used abundant knowledge-based features with fine-tuned SVM classifiers and achieved the F1 score of 70.2 and 67.2%, respectively. For a fair comparison, we also integrated the knowledge feature into the distant supervision paradigm and obtained the F1 score of 72.1%. This suggests that our method can effectively take advantage of the knowledge base features as well.

## Conclusions

This paper exhibits a distant supervision paradigm for the automatic chemical-induced disease relation extraction. The paradigm is built on an attention-based model and a stacked auto-encoder network model for intra- and inter-sentence relation extraction, respectively. Experimental results show that the attention mechanism considering various features of concepts and contexts is effective on intra-sentence relation extraction under distant supervision paradigm. Furthermore, its combination with the auto-encoder model at inter-sentence level achieves the best performance on the CID relation extraction task without direct application of KB.

We believe the success of distantly supervised CID relation extraction can be generalized to other relation extraction tasks in the biomedical literature. In future work, we intend to adopt dependency information for relation extraction in distant supervision paradigm, though this will bring about the heavy burden of dependency parsing. On the other hand, discourse structure will be explored to further improve the relation extraction performance at inter-sentence level.

## Abbreviations

CDR: Chemical-Disease Relations
CID: Chemical-induced Disease
CNN: Convolutional neural network
CTD: Comparative Toxicogenomics Database
DS: Distant supervision
IAA: Inter-Annotator Agreement
KB: Knowledge Base
LSTM: Long Short-Term Memory
ML: Machine learning
PPI: Protein-Protein Interaction
RNN: Recurrent Neural Network
SVM: Support Vector Machine
